# Soluble Papain to Digest Monoclonal Antibodies; Time and Cost-Effective Method to Obtain Fab Fragment

**DOI:** 10.3390/bioengineering9050209

**Published:** 2022-05-12

**Authors:** Matthew Collins, Hanieh Khalili

**Affiliations:** 1 School of Health, Sport and Bioscience, University of East London, London E15 4LZ, UK; m.collins@uel.ac.uk; 2 School of Pharmacy, University College London, London WC1N 1AX, UK; 3 School of Biomedical Science, University of West London, London W5 5RF, UK

**Keywords:** papain, IgG, enzymatic digestion, protein L purification

## Abstract

Antigen binding fragments (Fabs) used in research (e.g., antibody mimetics, antibody-drug conjugate, bispecific antibodies) are frequently obtained by enzymatic digestion of monoclonal antibodies using immobilised papain. Despite obtaining pure Fab, using immobilised papain to digest IgG has limitations, most notably slow digestion time (more than 8 h), high cost and limited scalability. Here we report a time and cost-effective method to produce pure, active and stable Fab using soluble papain. Large laboratory scale digestion of an antibody (100 mg) was achieved using soluble papain with a digestion time of 30 min and isolated yields of 55–60%. The obtained Fabs displayed similar binding activity as Fabs prepared via immobilised papain digestion. Site-specific conjugation between Fabs and polyethylene glycol (PEG) was carried out to obtain antibody mimetics FpF (Fab-PEG-Fab) indicating that the native disulphide bond had been preserved. Surface-plasmon resonance (SPR) of prepared FpFs showed that binding activity towards the intended antigen was maintained. We anticipate that this work will provide a fast and less costly method for researchers to produce antibody fragments at large scale from whole IgG suitable for use in research.

## 1. Introduction

**Figure 1:**
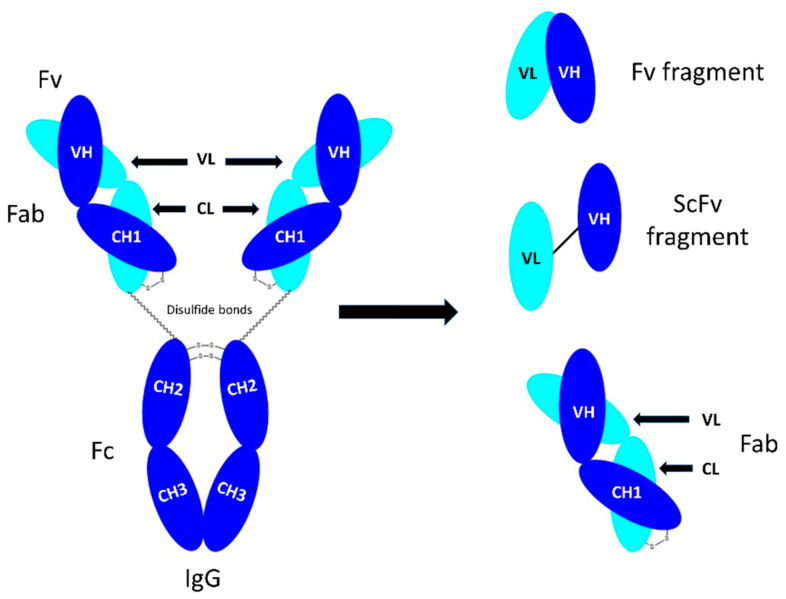
Representation of the structure of the IgG motif and related fragments. CH = constant heavy chain; Fab = fragment antigen binding; Fc = fragment crystallizable; Fv = variable fragment; scFv = single chain Fv.

An IgG antibody consists of four polypeptide chains including two identical heavy chains and two identical light chains (Figure 1). The light chains can be either κ (kappa), λ (lambda) or σ (sigma), based on differences in the amino acid sequence [1]. Each heavy (H) and light chain (L) contains a variable (V) and a constant (C) region (Figure 1). The variable regions are responsible for specificity and antigen binding affinity and contain approximately the first 110 amino acids [2] that form part of the fragment antigen-binding (Fab) region. The hyper-variable regions are complementary determining regions (CDRs). Fragment crystallisable (Fc) is a homodimer, consisting of the heavy constant (CH2), and CH3 domains (Figure 1). The CH2 and CH3 domains are covalently bound by disulphide bonds in the hinge region [3]. It is thought that the CH2 and CH3 domains are required for IgG catabolism [4]. The two Fabs and Fc are connected by a flexible hinge region.

When only the binding function of an antibody is required, such as blocking biological activity of molecules, or engaging a signalling pathway through cross-linking receptors, it is possible to utilise much smaller proteins known as antibody fragments that display high affinity binding properties. There is much interest in developing Fabs as a means to discover highly selective molecules [5,6,7] with three FDA-approved currently in the clinic such as abciximab (Reopro), idarucizumab (Praxbind) and ranibizumab (Lucentis). Anti-VEGF ranibizumab approved in 2006, has revolutionized treatment of age-macular degeneration (AMD) disease. In addition to therapeutic application, Fabs can be used in a wide range of protein conjugation, such as antibody-drug conjugates (ADCs), antibody mimetics, bispecific antibodies and radiolabel antibody fragments. Certolizumab pegol (Cimzia) is an anti-TNFa Fab that has been covalently conjugated to a polyethylene glycol (PEG) and used in treatment of rheumatoid arthritis. Bispecific faricimab (Vabysmo), recently approved in 2022 for treatment of AMD, is comprised of two Fabs (anti-VEGF and anti-angiopoietin 2) that are conjugated via Fc fragment. Hence, there is a need for an optimal method to prepare high amount of pure and stable Fabs with preserved antigen-binding activity.

While it is possible to engineer Fabs using bacterial expression systems, expression of Fabs containing essential disulphide bonds necessary for their activity and stability, is challenging using an *Escherichia coli (E. coli)* system [8,9]. Several limitations in yield, folding and functionality are also sometimes encountered in *E. coli* production of Fab fragments. An alternative is the preparation of a Fab fragment by enzymatic digestion of monoclonal antibodies [10,11,12]. Enzymatic digestion of IgG to obtain Fab, has long been studied using papain. Papain has a molecular weight of 23 kDa and was originally isolated from crude papaya (*carica papaya*) latex obtained from the unripe papaya fruit [13] and can cleave monoclonal antibodies above the hinge region to cleave the Fc fragment and to obtain Fab fragments. It is a thiol-endopeptidase with 212 amino acids that is stabilised with three disulphide bonds [13]. It has a sulphydryl group in the active site, which must be in the reduced form for papain to be proteolytically activated. While papain is a non-specific endopeptidase, other enzymes, such as gingisKHAN and FabULOUS, are very specific and can digest IgG at a single digestion site to obtain Fab. A drawback of these enzymes is their significantly higher cost (to digest 100 mg IgG, 200,000 units of gingisKHAN are needed at the cost of GBP 10,000), this can limit their use in research especially when large quantities of Fabs are required.

**Figure 2:**
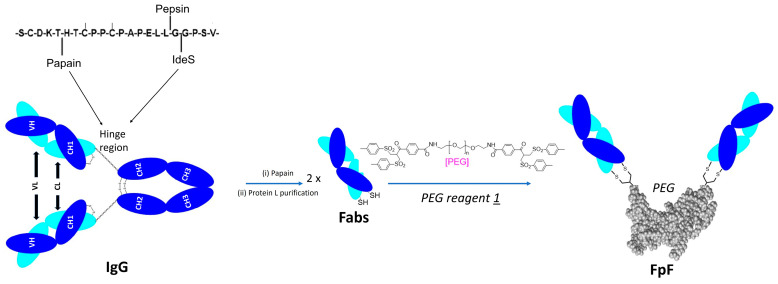
Preparation of FpF from Fabs and PEG reagent 1. Fabs are obtained from papain digestion of IgG.

Historically, enzymatic digestion of IgG was performed by soluble form of papain but several challenges were faced, such as purification and over digestion. These challenges led to development of immobilised forms of papain in which the enzyme is immobilised onto agarose beads, which aid with purification as enzymes are simply removed via centrifugation. Despite obtaining pure Fab, using immobilised papain to digest IgG has limitations, most notably slow digestion time (more than 8 h), and high cost which limit scalability within research settings as we also experienced in our optimized protocol [14]. Hence, we aimed to revisit the use of soluble papain and discover how to overcome challenges involved with purification and over digestion with a hope to (i) lower the cost, (ii) scale up digestion to 100 mg IgG and (iii) speed up the digestion process to less than 1 h. Here we also provided an example where prepared Fabs could be used to generate an antibody mimetic called Fab-PEG-Fab (FpF) (Figure 2) [15,16] as we previously developed.

Careful consideration had to be given to the purification method to purify the Fab from other fragments in the digestion mixture. While protein A was suitable for the immobilised papain protocol, it was not suitable for a methodology using soluble papain. This is because protein A binds to the Fc regions and cannot separate the soluble enzyme from the Fabs, leading to over digestion. In contrast, protein L binds to the kappa light chains located in the Fabs allowing the soluble enzyme and the Fabs to be separated effectively.

The stability and binding integrity of the purified Fab prepared from soluble papain was studied using SDS-PAGE analysis and surface plasmon resonance (Biacore assay). It was found that digestion time could be reduced to 50 min when soluble papain was used to digest 100 mg IgG. Different IgGs (humanised and chimeric) were studied in this paper, to investigate if they are digested differently using soluble papain. It was found that humanised IgG (e.g., tocilizumab anti-IL6R IgG1 and bevacizumab anti-VEGF IgG1) were digested using soluble papain with high recovery yield of 55–60% of purified Fabs. Chimeric IgG (e.g., infliximab anti-TNFa IgG1) was also digested by soluble papain but with a lower recovery yield. Digestion of Fc-fusion protein (e.g., aflibercept) using soluble papain resulted in the fragments which had no interchain disulphide needed for further conjugation processes. It was also not possible to use protein L chromatography for purification because no kappa light chain is present in a Fc-fusion protein. The binding of purified Fab_beva_ was maintained against VEGF_165_ using Biacore assay. Stability of the Fabs obtained by soluble papain was also maintained for duration of 5 months at −20 °C. Site-specific conjugation of Fabs obtained from soluble papain, were performed using PEG reagent 1 and resulting FpFs showed similar binding to FpFs prepared using the immobilised papain digestion process.

In this study, we have shown for the first time that soluble papain is used to digest 100 mg IgG of different antibody-based medicines (chimeric, humanised IgG and Fc-fusion protein) to obtain intact and stable Fabs in less than 1 h. Digestion with soluble papain is a cost reducing methodology that we suggest as a replacement for immobilised papain digestions. When treating purification and reagent costs as equal, digestion of 100 mg IgG with soluble papain can be up to 90 times less than if immobilised papain was used for the same process.

## 2. Material and Method

### 2.1. Materials

Bevacizumab (Avastin, 25 mg/mL, Genentech, South San Francisco, CA, USA) and tocilizumab (Actemra, 20 mg/mL, Roche, Basel, Switzerland) were purchased from a pharmacy for research purposes. Infliximab and aflibercept were obtained from the pooled remains of vials that had been used clinically. Soluble papain, immobilised papain, protein L (Hitrap Protein L 5.0 mL) column, NAb protein-A spin columns, elution immunoPure IgG buffers (0.1 M glycine, pH 2.8), neutralization buffer (1.0 M Tris buffer, pH 8.5), binding buffer (1.0 M Tris buffer containing EDTA, pH 8.0), Novex Bis-tris 4–12% gels, Sharp blue standard protein markers, NuPAGE MOPS running buffer were purchased from Thermo Fisher (Pierce, MA, USA). Phosphate buffered saline (PBS) containing NaCl (0.16 M), KCl (0.003 M), Na_2_HPO_4_ (0.008 M) and KH_2_PO_4_ (0.001 M) was prepared with tablets purchased from Oxoid (Hampshire, UK). InstantBlue Coomassie stain was purchased from Abcam (Cambridge, UK). PD-10 columns, cation exchange columns (HiTrap SP HP 1.0 mL) and a Superdex 200 prep grade size exclusion column (34.0 µm particle size) along with Biacore consumables including immobilisation reagents and buffers: N-hydroxysuccinimide (NHS), 1-ethyl-3-(3-dimethylaminopropyl)-carbodiimide (EDC), ethanolamine-HCl (1.0 M, pH 8.5), glycine buffer (10 mM, pH range of 1.5–2.5), HBS-EP buffer and sensor chips, were all purchased from GE Healthcare (Chicago, IL, USA). Human vascular endothelial growth factor (VEGF_165,_ 10 μg), sodium phosphate monobasic monohydrate (NaH_2_PO_4_), sodium phosphate dibasic (Na_2_H_2_PO_4_), ethylenediaminetetraacetic acid calcium disodium salt (EDTA), L-cysteine and cysteine hydrochloride were purchased from Sigma-Aldrich (St. Louis, MO, USA). Protein L chromatography was conducted using an AKTA prime plus LC system. Size exclusion was conducted using an AKTA purifier.

### 2.2. Methods

Digestion using immobilised papain was conducted following the optimised method reported in [14]. For soluble papain, digestions at a scale of 100 mg were optimised as following:

Digestion buffer was first prepared by dissolving cysteine (50 mM L-cysteine) in the phosphate buffer (20 mM Na_2_H_2_PO_4_, 10 mM EDTA) and pH was then adjusted at pH 7.0. Lyophilised soluble papain (5.0 mg) was dissolved in digestion buffer (1.0 mL) to prepare soluble papain solution (5 mg/mL).

One-hundred milligrams tocilizumab (20 mg/mL, 5.0 mL) was diluted with digestion buffer (34 mL) and the soluble papain (5.0 mg/mL, 1.0 mL) was then added to the diluted tocilizumab and incubated at 37 °C for 30 min. After 30 min, the digestion mixture was removed from the incubator and immediately injected onto a protein L column (5.0 mL HiTrap Protein L) which was connected to an AKTA prime plus system. The column was equilibrated with binding buffer (100 mM sodium phosphate, 150 mM sodium chloride, 500 mL, pH 7.2) prior to digestion. The unbound components within the digestion mixture (Fc fragments, papain and L-cysteine) were allowed to elute from the column using a flow rate of 2 mL/min and collected in flow through fractions. Once the digestion mixture is injected onto the column the digestion ceases as the Fab is no longer in contact with the soluble enzyme. It is key to inject the digestion mixture immediately to limit over digestion and maximise yield. For this reason it is not possible to characterise the digestion mixture using SDS-PAGE analysis. Once the UV signal had returned to baseline fab elution buffer (100 mM glycine, pH 2.5) was used to elute the bound Fab fragments from the column at a flow rate of 2 mL/min and elution fractions collected. To elute the fab fragments the concentration of elution buffer was set to 100% immediately, a gradient was not used. Size-exclusion chromatography (SEC, 24.0 mL Superdex 200 10/300 GL) was used to further purify the fab fractions at a flow rate of 0.5 mL/min, using phosphate buffered saline (PBS) as the mobile phase. Fractions of purified Fabs were collected during SEC and analysed using SDS-PAGE analysis.

Digestions starting with 100 mg of tocilizumab typically gave 30–35 mg of Fab_tocili_ after purification, a 50–55% approximate yield. Site-specific conjugation was carried out using the protocol established and reported in [14]. Anti-VEGF molecules (bevacizumab, Fab_beva_ and FpF_beva_) were selected as an example to study their binding activity to VEGF using Biacore X-100 (GE Healthcare, Chicago, IL, USA). A CM3 chip was immobilised with VEGF_165_ (95 RU) using an amine-coupling immobilisation method, reported in [11].

## 3. Results and Discussion

To initiate IgG digestion, papain must be in the reduced form to be proteolytically activated. Therefore, cysteine is added to the papain preparation to activate the enzyme. EDTA is included in the digestion buffers to chelate with metals, such as copper, that can catalyse the reoxidation of reduced papain. The pH of the digestion buffer was adjusted to 7.0 since this is the optimal pH for the enzymatic activity of papain, a temperature of 37 °C was also used as it is also optimal for enzymatic activity. Immobilised papain consists of the enzyme being immobilised onto an agarose resin which takes the form of a slurry. This aids purification because after digestion the only protein species in solution are those derived from the antibody, simply, the slurry can be centrifuged and separated from the digested antibody. It is, however, very expensive and the digestion process is time-consuming. Alternative forms of papain, i.e., soluble papain in a crude or lyophilised form, are available at much lower cost but with greater purification challenges.

**Figure 3:**
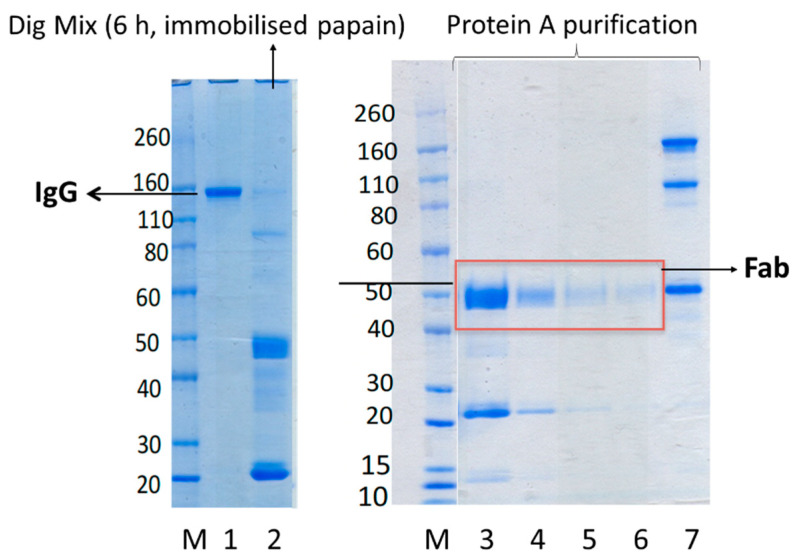
Representative SDS-PAGE gel of digestion of humanized IgG (bevacizumab in this gel) using immobilised papain (lanes 1 and 2) and then purification using protein A column (lanes 3–7). Novex Bis-Tris 4–12% gel stained with colloidal blue (lanes M–7). Lane M: protein standard marker, lane 1: bevacizumab before digestion, lane 2: bevacizumab digestion mixture after 6 h incubation with immobilised papain at 37 °C, lanes 3–6: four flow through fractions collected from protein A columns using binding buffer which contain the purified Fab_beva_. Lane 7: elution fraction containing Fc and undigested bevacizumab and intermediate fragments such a F(ab’)_2_.

We had previously optimised digestion conditions using immobilised papain [14] to ensure the antibody would be completely digested to produce the Fab and Fc fragments while ensuring the binding sites of the Fab were not damaged [14,15,16] (Figure 3). IgG was fully digested after 6 h of digestion. Shorter incubation time resulted in partial digestion of IgG and lower yield of Fab. Furthermore, because of the cost associated with immobilised papain, it was not cost-effective to digest more than 20 mg IgG. Hence, we wanted to examine if soluble papain available at much lower cost (100 mg soluble papain for GBP 110, is enough to digest 2000 mg IgG vs. 5 mL immobilised papain for GBP 270, which is enough to digest 50 mg IgG) could “cleanly” digest IgG (at scale of 100 mg) and prepare pure, and stable Fabs which maintain binding to their target antigens.

Soluble papain is available in both crude and lyophilized forms. While we initially used both forms of soluble papain, it was then thought to continue using lyophilised papain because of the impurities present in crude papain [17,18]. Some of the components in crude papain are not water-soluble which lead to the method having poor reproducibility. In addition, the proteolytic activity of the crude (un-purified) papain is one-fourth to one-half that of the crystalline papain [19]. During initial experiments with soluble papain it was found that visualising the digestion mixture by SDS-PAGE was not possible, this contrasts with digestions using immobilised papain. This was due to not being able to remove soluble papain from the digestion mixture to stop the digestion. Freezing and pH adjustment of the digestion mixture were assessed to stop the digestion, however, this was not successful. To remove the soluble papain from digestion mixture to stop the unwanted digestion and also purify the Fab fragment, the protein L purification method was applied.

**Figure 4:**
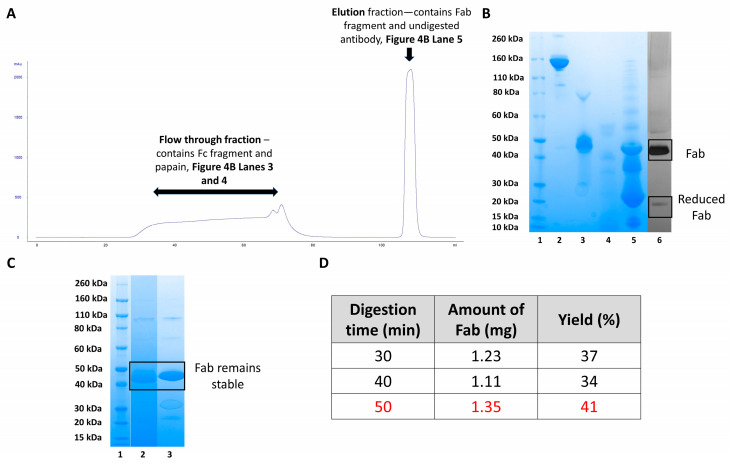
(**A**) Annotated protein L chromatogram for the purification of a 100 mg tocilizumab digestion mixture, using soluble papain. (**B**) SDS PAGE analysis of flow through and elution fractions from the protein L purification. Novex Bis-Tris 4–12% gel stained with colloidal blue, lane 1: standard marker, lane 2: parent tocilizumab, lanes 3 and 4: flow through fractions containing Fc fragment and papain, they were collected in two parts due to the size of the column, lane 5: elution fraction containing Fabs and undigested IgG, lane 6: silver-staining of the purified Fab after SEC, reduced-Fab at 25 kDa observed due to presence of cysteine in the digestion buffer. (**C**) SDS-PAGE analysis of purified Fab_tocili_ after 5 months storage at −20 °C. Novex Bis-Tris 4–12% gel stained with colloidal blue. Lane 1: protein standard marker, lane 2: Fab_tocili_ at initial timepoint, lane 3: Fab_tocili_ at 5-month timepoint after storage at −20 °C, no light/heavy chain dissociation or degradation observed. (**D**) Table showing the impact of digestion time on yield when using soluble papain, yield calculated using UV visible spectroscopy at a wavelength of 280 nm.

While protein A column was used initially to purify Fab, but it was not suitable as the enzyme and the desired Fab regions were not separated. This led to the further digesting of the Fabs and results in small, unusable antibody fragments with little function. Instead, protein L column was used to purify Fabs from the digestion mixture. Protein L successfully purified and maintained the Fabs because it specifically binds to the kappa light chain of the Fabs allowing separation from the enzyme as indicated in Figure 4A,B. When using protein L, the unbound Fc regions, soluble papain and cysteine do not bind to the column bed and elute during column flow through (Figure 4A chromatogram—28–87 mL and Figure 4B SDS-PAGE lanes 3 and 4). Fabs and other fragments containing kappa light chains interacted with the protein L column and eluted during the elution step (Figure 4A chromatogram—104.64–112 mL and Figure 4B SDS-PAGE lane 5). Further purification of the elution fraction was carried out using SEC to obtain pure final Fab fragments suitable for conjugation (Figure 4B, lane 6). Silver-staining on the purified Fab indicated no impurity, a band displayed in 50 kDa corresponded to the Fab and a band in 25 kDa molecular weight, corresponded to the reduced-Fab as a result of using cysteine in the digestion buffer. To study the quality of the purified Fabs, stability studies were carried out for a duration of five months, As in Figure 4C, lane 3 shows that the Fab maintained its structure with no degradation or light and heavy chain dissociation after being stored at −20 °C for five months.

Figure 4D shows a table summarising different digestion times and the amount of Fab obtained presented as the final yield. Fast digestion times of less than one hour were achieved using soluble papain compared to immobilised papain (6–8 h digestion time). It was also found that a digestion time of 50 min gave the greatest amount of purified Fab, compared with 30- and 40-min digestion, yield was calculated by UV-visible spectroscopy at 280 nM. For the digestion of 100 mg of tocilizumab, a 30-min digestion time was chosen. This was because the large volume of digestion mixture (40 mL) must be applied to the protein L column over a period of time. Application of the digestion mixture at the chosen flow rate of 2 mL/min resulted in total digestion time of 50 min.

**Figure 5:**
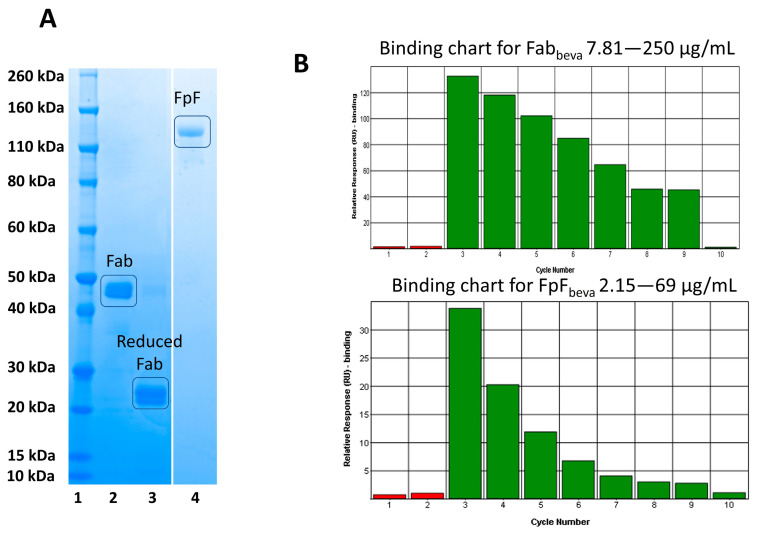
(**A**) SDS-PAGE gel of site-specific conjugation of obtained Fab from soluble papain digestion of IgG, Novex Bis-Tris 4–12% gel stained with colloidal blue (lanes 1–4). Lane 1: protein standard marker, lane 2: purified Fab_beva_ after protein L and SEC chromatography, lane 3: reduced Fab_beva_; Fab_beva_ was incubated with DTT for 30 min, and excess DTT was removed by PD-10 column, lane 4: purified FpF_beva_ which resulted from site-specific conjugation of two Fabs with PEG reagent 1, (**B**) binding assay using SPR, A CM3 chip was immobilised with VEGF165 (95 RU immobilization level), Fab_beva_ and FpF_beva_ were applied over functionalised chip. Binding to VEGF was maintained for both Fab_beva_ and FpF_beva_.

To ensure the obtained Fabs from soluble papain enzymatic digestions were capable of undergoing site-specific conjugation, an FpF antibody mimetic was prepared and its binding activity was evaluated. The Fab interchain disulphide was first reduced using a reducing agent (DTT) (Figure 5, lane 3). The excess DTT was then removed using a PD-10 column and 1 molar equivalent of PEG reagent 1 was added. The resulting FpF was purified using ion-exchange chromatography with the purified FpF being shown in Figure 5, lane 4.

Binding affinity of Fab_beva_ obtained from bevacizumab (anti-VEGF IgG) and conjugated Fab_beva_ (FpF_beva_) was then examined using SPR, as seen in Figure 5B. These binding charts suggest that the binding of the Fab_beva_ and FpF_beva_ were maintained in a concentration dependent manner.

**Figure 6:**
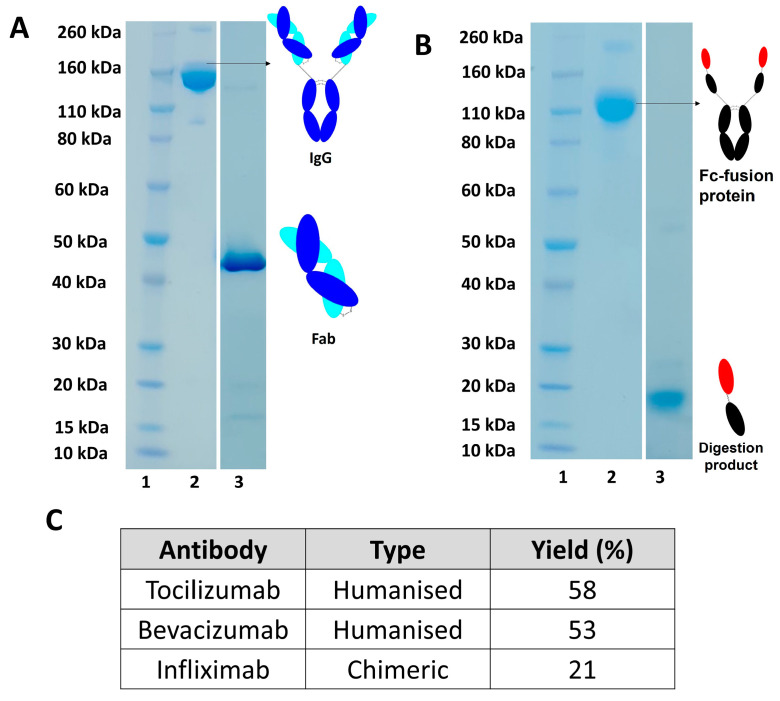
(**A**,**B**) SDS-PAGE gel of digestion of different antibody-based medicines, (**A**) monoclonal antibody, (**B**) Fc-fusion proteins, Novex Bis-Tris 4–12% gel stained with colloidal blue (lanes 1–3). Lane 1: protein standard marker, lane 2 (**A**) IgG before digestion, lane 3 (**A**): purified Fab after protein L and SEC chromatography, lane 2 (**B**) Fc-fusion protein, lane 3 (**B**) digestion product after protein A purification, without any interchain disulphide bond, (**C**) table to compare digestion yield for humanised and chimeric IgGs, concentrations were calculated using UV-Visible spectroscopy at 280 nm.

Digestion of different monoclonal antibodies (humanised and chimeric) using soluble papain were also examined, as shown in Figure 6A with yields calculated using UV-visible spectroscopy as shown in Figure 6C. Papain digestion of humanised IgG, such as bevacizumab and tocilizumab, resulted in a higher yield of Fab compared to digestion of a chimeric IgG (e.g., infliximab), as seen in Table C, Figure 6. Digestion of an Fc-fusion protein, such as aflibercept, resulted in a fragment that was not suitable to for further conjugation as cleavage occurred after hinge region leading to the preparation of a fragment with no interchain disulphide bond (Figure 6B).

## 4. Conclusions

For therapeutic and diagnostic applications, as well as basic research, it is vital to produce Fab fragments in large amounts with reserved binding activity. Historically, Fab fragments are prepared by papain digestion of whole antibody molecules. While using immobilised papain to digest IgG, pure and stable Fabs can be obtained; however, the method is slow and unscalable in a research setting due to excessive cost. Within this study we developed a robust and reproducible research method to obtain high amounts of homogenous Fab in a short period of time using a cheaper soluble form of papain. The obtained Fab possessed binding activity towards its antigen and preserved its stability during storage condition. The Fabs are also suitable for the further preparation of novel antibody formats for research purposes.

## Data Availability

Data available in a publicly accessible repository that does not issue DOIs.
